# Supplementary material for: Are carbon emissions trading and green financial instruments synergistic? -Comprehensive quantitative research based on content analysis
Source: PLoS One. 2024 Mar 7;19(3):e0298601. doi: 10.1371/journal.pone.0298601 (PMC10919601; doi:10.1371/journal.pone.0298601)
Supplement: S1 Data — (ZIP) [file pone.0298601.s001.zip › Data/地级市Z_F工作报告.zip!/║■─╧/╥μ╤⌠╩╨/└┤╫╘╣1⁄2╓┌║┼í╛┬φ┐╦╩2╛▌═°í┐_2012.docx]

各位代表：

　　现在，我代表市人民政府向大会作工作报告，请予审议，并请市政协各位委员和其他列席同志提出意见。

　　一、2011年工作回顾

　　2011年是“十二五”的开局之年。一年来，我们在中共益阳市委的正确领导下，团结带领全市人民，按照“坚持科学发展，奋力后发赶超，建设绿色益阳”的总体思路，强力推进园区建设和交通建设两大会战，不断深化改革，努力改善民生，实现了经济的平稳较快发展和社会大局的和谐稳定。全年完成生产总值880亿元（预计数，下同），比上年增长13%，其中第一、二、三产业增加值分别增长3%、17.5%和14.3%；完成财政总收入60亿元，同口径增长32.8%；完成固定资产投资452亿元，增长33%；实现社会消费品零售总额306亿元，增长18%；城镇居民人均可支配收入17446元，增长13.3%；农民人均纯收入6515元，增长16%。年末金融机构各项存款余额725.7亿元，比年初增加117.2亿元；各项贷款余额427.8亿元，比年初增加67.8亿元，比上年多增22.2亿元。

　　（一）三次产业协调发展

　　积极推动产业结构调整，三次产业结构比由上年的22.8:40.5:36.7调整为22.5:42.2:35.3，第二产业比重提高1.7个百分点。

　　农业经济稳步发展。农林牧渔业实现总产值285亿元，比上年增长3%。粮食连续8年增产，蔬菜、茶叶、水产等产量和产值均有增长。农产品加工业实现增加值91.5亿元，比上年增长22%。农村土地流转面积达252万亩，其中试点乡镇土地信托流转13.63万亩。组织战胜了60年一遇的春夏秋连旱灾害，成功抗御了6月份连续五轮强降雨的侵袭。出台了《益阳市水利建设十大工程实施方案》，全年共投入水利建设资金12亿元，水利建设取得新的成效。全面完成了环洞庭湖基本农田建设重大工程年度任务。

　　工业经济增势强劲。全市工业总产值突破1000亿元；实现工业增加值335亿元，比上年增长17.8%，其中规模工业增加值310亿元，增长22%，增速排名全省第一；工业对经济增长的贡献率达50%。新进规模工业企业78家，全市规模工业企业达782家。装备制造、食品加工、电子信息等十大优势产业实现增加值270亿元，比上年增长21.5%。高新技术产业实现增加值68.9亿元，增长35.7%。继续培育优势品牌，新增中国驰名商标4个、湖南名牌4个。建筑业实现增加值36.1亿元，比上年增长14.5%。

　　第三产业较快增长。实现第三产业增加值311亿元，比上年增长14.3%。旅游业较快发展，安化茶马古道、山乡巨变第一村成功创建国家4A级景区，皇家湖生态旅游度假区基本建成并对外试营业。房地产业健康发展，全年完成房地产开发投资75亿元，实现商品房销售面积235万平方米，商品房销售额58亿元，比上年分别增长21%、20.9%和34.9%；成功举办了第三届房交会。

　　（二）项目建设成绩突出

　　园区建设大会战效果初显。益阳高新区升格为国家级高新区。九大工业园区全年完成投资218亿元，签约落户项目169个；实现工业增加值175亿元，比上年增长69.9%，其中规模工业增加值168亿元，增长66.2%；实现税收19.6亿元，增长76.5%；园区工业增加值占全市工业增加值的比重达到52.2%。

　　交通建设大会战扎实开展。全年完成交通建设投资50.1亿元。石长铁路复线益阳段，常安、安邵、岳常高速益阳段，319国道益阳南线高速公路等在建项目向前推进。G207线益阳段、S205桃江段一期基本建成；S308桃马公路、S225安化段、益阳港泥湾港区进港公路、沙头资江大桥、沅江黄茅洲大桥等项目正在抓紧建设。完成农村公路建设961公里，建成农村客运站6个。朝阳汽车站、益阳港泥湾港区千吨级码头相继建成。

　　产业项目建设来势喜人。全年完成工业投资326.1亿元，比上年增长37.6%。三一中阳产业园、森华林业人造板生产线、奥士康精密线路板生产线一期、金沙重机年产40万吨型钢生产线、圣德锰业电解锰等项目竣工投产。国晶硅业、沅纸20万吨化机浆生产线、科力远动力电池新厂、海螺水泥、南方水泥二期、拓普竹麻等项目顺利推进。

　　（三）城镇化水平稳步提升

　　城市创建全面推进。创建国家卫生城市工作深入推进。创建全国双拥模范城通过验收。成功创建为省级文明城市和中国优秀旅游城市。创建国家森林城市工作取得阶段性成果，“六大创森工程”扎实推进。创建全国文明城市工作全面铺开。创建国家交通管理模范城市全面启动。

　　城镇建设成绩突出。中心城区完成基础设施建设投资32亿元。完成了11条主次干道和214条背街小巷的提质改造。超额完成了第三个“十万株树进城”任务，改造和新建社区公园4处、街头绿地40处。完成了城区重要地段绿化亮化、五里堆片区雨污处理工程等项目。中心城区农贸市场提质改造取得新的进展。城市经营迈出新的步伐，市城建投全年完成城市建设筹融资8.75亿元。小城镇建设扎实推进。桃江、沅江、大通湖、安化城镇生活垃圾无害化处理设施建成运行；沧水铺镇成功创建全国文明村镇和省级卫生镇；灰山港、梅城、迎风桥、茅草街等一批重点镇建设步伐进一步加快；草尾镇作为统筹城乡发展试验镇，探索建立了农村居民集中居住新模式。全市城镇化率达到42%，比上年提高1.6个百分点。

　　城镇管理得到加强。加大市容环境整治力度，重点整治中心城区“脏、乱、差”和公共场所“五小”卫生问题，城区卫生保洁实现全覆盖、常态化。集中开展渣土运输、户外广告、建设工地文明施工等专项整治行动，市容市貌有新的改善。开展“城管进社区”工作，创新“门前三包五不准”责任落实办法，启动“数字城管”精细化管理，城市管理规范化、社会化水平不断提高。着力加强城市社区建设，社区社会管理服务功能明显增强。

　　（四）绿色益阳建设扎实有效

　　节能减排成效明显。全市万元生产总值综合能耗比上年下降3%，万元规模工业增加值能耗下降7.5%。加大污染减排工作力度，实施减排项目44个，化学需氧量、二氧化硫排放量分别削减1.7%、1.8%；全市城镇生活污水处理率达到82%。我市污染减排工作受到省政府表彰。

　　环境保护和生态建设得到加强。加强城乡环境综合整治，突出抓了有色金属行业、危险化学品行业、医药行业和环洞庭湖区域重点污染企业的污染防治。大力实施农村清洁工程，农村环境卫生状况明显改善，益阳被列为全省农村环境综合整治试点示范城市。大力加强生态建设，全市森林覆盖率达到54%。资阳区被列为国家级生态示范区，赫山区被批准为国家级生态旅游示范区，南县被评为国家生态农业县；安化柘溪成为国家湿地公园，六步溪成为国家级自然保护区。益阳被评为中国杰出绿色生态城市。

　　“两型”社会建设加快推进。益阳市“两型”社会建设总体实施方案和益阳东部新区改革建设实施方案、片区规划、土地利用规划先后获得省政府批准。鱼形山“两型”社会示范区招商引资初见成效，与省两型办、省广播电视台、省体育产业集团、御邦投资公司、山东兖矿集团等签订了合作开发的框架协议。

　　（五）改革开放不断深化

　　各项改革稳步推进。政府机构改革不断深化。行政审批制度改革深入推进，行政许可项目和非行政许可项目分别减少16.5%和18.4%。政务中心建设不断加强，公共资源交易中心规范高效运行。文化体制改革正在推进。国企改革继续深化。行政事业单位国有资产管理与经营进一步规范。卫生体制改革顺利推进，国家基本药物制度实现全覆盖，农村和社区所有医疗卫生机构全部实行基本药物零差率销售。集体林权制度改革、水务管理体制改革等有效推进。

　　开放引资取得实效。招商引资成效明显，全年引进市域外资金252.5亿元，比上年增长23.4%，形成固定资产投资205亿元，其中引进境内省外资金142亿元，增长26.9%，直接利用外资1.2亿美元，增长37.1%。立项争资取得新的成绩，全年争取各类资金106.2亿元，比上年增长15.4%。招“行”引资取得突破，交通银行益阳分行挂牌营业，华融湘江银行益阳分行获批筹建；全市新增融资性担保公司2家、小额贷款公司1家；上海复星集团和深圳达晨创投分别入股天运林工和益华水产，明星麻业在天津股交所实现股权融资。

　　对外贸易较快增长。全年完成进出口总额4.8亿美元，增长28%，其中出口3.9亿美元，增长20%。全市出口额超过1000万美元的生产企业达到6家。

　　（六）民生工程成效明显

　　积极推进为民办实事工作。为民办实事32项省定目标任务全面完成和超额完成。全市新增城镇就业3.58万人，新增农村劳动力转移就业5.61万人。五大社会保险新增参保人数144万人，比上年增长44%；收缴各类保费21.3亿元，增长16%。保障城乡低保对象29.61万人、五保对象4.21万人，发放各类社会救助资金5.32亿元，比上年增长32.5%。改扩（新）建乡镇敬老院13所。新增廉租住房9409套，完成农村危旧房改造6693户。完成了14个乡镇邮政局所补建和146个村电网新建改造任务。完成了17座小Ⅰ型病险水库的除险加固，解决了农村24.76万人的饮水安全问题。减负惠农政策、城乡低收入群体价费优惠政策等落实到位。扶贫和移民后扶工作力度加大，区县（市）对口帮扶安化贫困乡镇和市直单位联村帮扶工作取得实效。

　　努力保障人民群众生命财产安全。扎实开展煤矿、非煤矿山、道路交通运输、水上安全和烟花爆竹五大专项整治行动，排查治理各类安全隐患近万处，督促企业投入安全生产整改、技改资金1.2亿元。严厉打击非法违法生产经营行为，关闭生产经营单位319家。全年安全生产事故死亡人数和较大事故均控制在省定目标以内。

　　（七）社会建设和管理扎实推进

　　社会事业全面发展。加快教育强市建设，“普九”成果进一步巩固，建成义务教育合格学校70所、农村公办幼儿园9所，职教资源整合工作得到加强。科技创新能力不断提升，省级工程技术研究中心增加到5个，6项科技成果获省科技进步奖，年专利申请量突破1000件。积极发展文化事业，新建乡镇综合文化站35个、农家书屋659家、社区文化活动室17个。加快体育事业发展，成功承办湖南省第五届农运会，我市代表团取得优异成绩。人口计生工作不断加强，连续6年保持全省先进。医疗卫生服务体系逐步健全，疾病防控能力进一步增强。爱国卫生工作有效开展，城市卫生位居全省前列。工商行政管理、食品药品安全监管和质量技术监督工作成效明显。国土资源管理取得新的成绩，全年共批回建设用地463.75公顷，基本保障了重点项目建设的用地需求。国防动员和后备力量建设扎实推进，全民国防教育不断加强。建设了益阳市电子政务外网平台，实现了省市县三级互联互通。益阳日报、益阳电视台、新闻出版、统计、档案、住房公积金、民族、宗教、外事、侨务、妇女儿童、老龄、台办、科协、地方志、消防、气象、水文、残疾人、红十字会等各项工作取得新的成绩。

　　精神文明建设得到加强。扎实开展“市民素质提升年”活动和第二届道德模范评选表彰活动，市民思想道德素质和城市文明程度有新的提高。以“文明创建进农家”和创“十星”户、“五星”村为载体，推动了农村文明村镇创建活动的深入开展。

　　社会大局和谐稳定。着力推进社会管理创新，部署了“法治益阳”建设任务，启动了“六五”普法工作，在全省率先建立了“特殊涉毒人员收治中心”和交通事故损赔纠纷联调联处机制。深入开展“三打一整”、“百日攻坚”、“清网行动”等专项整治行动，有效打击各类违法犯罪行为。坚持用群众工作统揽信访工作，切实加强人民调解、司法调解、行政调解“三调联动”，加大领导干部接访包案力度，信访渠道进一步畅通，赴省进京非正常上访批次明显减少。严格落实综治维稳工作责任制，全面实行重大事项社会稳定风险评估，强力推行综治维稳工作“村为主”模式，及时排查、化解与处置了一系列不稳定因素，确保了社会大局和谐稳定。综治工作被评为全省先进。

　　（八）政府自身建设得到加强

　　以市长热线、党风廉政舆情在线、人民网地方领导留言板、信访接待和人大代表建议、政协委员提案为主通道，主动对接老百姓利益诉求，解决了一些事关群众切身利益的实际问题；全面推进政务公开，优化行政审批流程，转变机关工作作风，政府机关行政效能得到提升；认真贯彻落实《湖南省行政程序规定》，坚持依法行政，健全政府科学民主依法决策机制和行政执法责任制、过错追究制，自觉接受人大及其常委会的法律监督、工作监督和政协的民主监督，自觉接受人民群众的监督；加强对重要部门、重点领域和关键环节的行政监察和审计监督，健全和落实行政问责制及相关配套制度，努力以清正廉洁的政风带动和促进各项工作。

　　各位代表，过去一年取得的成绩来之不易。这是中共益阳市委正确领导的结果，是市人大、市政协监督和支持的结果，是全市人民开拓创新、团结拼搏的结果。在此，我代表市人民政府，向辛勤工作在各行各业的广大干部群众，向各位人大代表、政协委员，向离退休老同志，向各民主党派、工商联、无党派人士和各人民团体，向中央和省属驻益单位，向驻益人民解放军、武警官兵和政法公安干警，向所有关心、支持和参与益阳改革发展的各界朋友，表示衷心的感谢！

　　在肯定成绩的同时，我们也清醒地认识到经济社会发展面临的困难和问题。主要是：经济总量偏小，经济结构不优，转型发展压力较大；财政规模小，收支矛盾十分突出；保障和改善民生任重道远，群众在上学、就业、就医、住房、养老等方面的实际困难仍然较多；影响和谐稳定的因素依然存在；机关工作作风需要继续改进，办事效率有待进一步提高。对这些问题，我们将高度重视，在今后的工作中采取有效措施认真加以解决。

　　二、2012年工作的指导思想和奋斗目标

　　2012年，是落实“十二五”规划的关键之年，是新的机遇与挑战并存的一年。从国际看，世界经济增长放缓，国际贸易增速回落，国际金融市场剧烈动荡，欧洲主权债务危机持续升级，世界经济形势总体上仍将十分严峻复杂，经济复苏的不稳定性、不确定性上升。从国内看，尽管受系统性风险增大、要素资源成本上升、投资和出口双回落等不利因素影响，经济增长速度温和下降，但经济环境仍将保持总体向好；中央经济工作会议确定了2012年经济社会发展“稳中求进”的工作总基调，国家将继续实行积极的财政政策和稳健的货币政策，增强宏观调控的针对性、灵活性、前瞻性，继续加大对“三农”、保障性住房、社会事业等领域的投入，继续支持欠发达地区、科技创新、节能环保、战略性新兴产业、国家重大基础设施在建和续建项目、企业技术改造等。从我市看，通过持续扩投资、打基础，全市基础设施条件正在改善，一批重大产业项目加速成长，对要素资源的吸纳能力不断增强，环省会比较优势和后发优势逐步显现，特别是益阳东部新区纳入“两型”社会建设示范区，将为我市对接和融入长株潭、加速崛起提供更多的战略契机。因此，我们一定要科学研判形势，坚定发展信心，抢抓发展机遇，积极应对各种困难和挑战，在新起点上努力开创后发赶超的新局面。

　　2012年政府工作的指导思想是：坚持以邓小平理论和“三个代表”重要思想为指导，深入落实科学发展观，全面贯彻党的十七届六中全会精神、省市党代会精神，紧紧围绕“坚持科学发展、奋力后发赶超、建设绿色益阳”的总体思路，突出抓好园区建设和交通建设两大会战，深入推进改革开放，着力改善民生民利，努力实现经济社会又好又快发展，不断提高人民群众的幸福指数。

　　2012年我市经济社会发展的主要预期目标是：生产总值增长13%，财政总收入增长20%，固定资产投资增长25%以上，社会消费品零售总额增长17%，规模工业增加值增长20%以上，城镇居民人均可支配收入增长12%以上，农民人均纯收入增长12%以上，城镇登记失业率控制在4.2%以内，万元生产总值能耗下降3%，人口自然增长率控制在6.8‰以内。

　　三、2012年工作任务

　　（一）强力推进园区建设大会战，助推新型工业化

　　强化园区兴工。把园区建设大会战作为推进新型工业化的重中之重。尽快出台加快园区发展的意见，建立健全市县联动的园区建设大会战工作机制。理顺中心城区园区管理体制，以益阳高新区为龙头，实行项目空间布局和规划建设统一管理。逐步将园区调度管理范围扩大到益阳船舶产业园、兰溪粮食产业园及重点乡镇工业小区，并严格目标管理考核。完善园区优惠政策，优化园区发展环境，强化各区县（市）对园区建设的领导与管理。力争园区工业增加值占全市工业增加值的比重达到55%以上，新建标准化厂房30万平方米以上。

　　大力培育优势产业。围绕先进装备制造、食品加工、电子信息、新材料、竹木加工等优势产业，扶持骨干企业发展，培育产业龙头。加快培育大企业大集团，增强对产业发展的集聚与带动能力。重点支持中联重科、三一中阳、国晶硅业、科力远等企业做大做强，大力支持有条件的企业上市。争取桃花江核电站早日开工建设。大力发展中小企业，增强协作配套功能。认真落实支持小微企业发展的各项政策。建立专业创业园区，统一提供基础设施、技术创新、产品销售、金融融资等服务平台，引导中小企业入园。设立中小企业发展专项资金，加强对中小企业的财税、信贷扶持，促进中小企业快速发展。

　　积极推进自主创新。强化企业自主创新的主体地位，引导企业加强与高等院校、科研院所的联系，开展多种形式的产学研合作。支持清华科技园和中南大学博云科技园建设。加快创新平台建设，进一步扶持市科技成果转化服务中心、国家专利技术长沙展示交易中心益阳分中心建设，加速科技成果向现实生产力转化。加强企业家队伍建设，引导和支持企业创建中国驰名商标和湖南省著名商标。

　　加快发展现代服务业。努力壮大文化旅游、商贸流通、房地产等优势产业，加快发展服务外包、现代物流、现代金融、信息服务等生产性服务业，全面提升交通运输、餐饮住宿、健身休闲、家政服务等生活性服务业。把旅游业作为支柱产业来培育，促进旅游与文化等相关产业的融合发展。以益阳高新区服务外包产业园为主平台，加快惠普、搜空高科等项目建设，重点引进培育一批呼叫中心、电子商务、动漫制作项目，力争5年内服务外包产业年营业额达到50亿元，从业人员突破1万人，成为全国“服务外包示范城市”。

　　（二）强力推进交通建设大会战，加快项目建设

　　狠抓交通基础设施建设。把交通建设大会战作为突破发展瓶颈的重中之重，确保全年完成投资70亿元以上。确保S308桃马公路等2条干线公路建成通车；加快推进岳常高速、常安高速、安邵高速益阳段和319国道南线高速公路、石长铁路复线益阳段、S225安化段、桃江县城绕城线、沙头资江大桥、沅江黄茅洲大桥、南茅生态运河改造等项目建设；全面启动益桃线拓改工程；确保益马高速、益娄高速益阳段、马安高速全面实质性开工，益南高速举行开工动员；完成益阳汽车北站提质改造，建成安化城南汽车站、南县南洲汽车货运站、南县南洲港区码头，启动公交停保场建设项目。同时，加快推进重大产业项目建设；确保民生工程顺利实施。

　　抓好立项争资工作。找准立项争资的切入点和着力点，在“三农”、保障性住房、节能环保、企业技术改造等方面，精心策划、包装、储备一批事关全局、带动力强的新项目。把立项争资工作列为绩效评估的重要指标，制定专项考核办法，细化分解目标任务，力争立项争资突破130亿元。

　　优化项目建设环境。优化政务服务环境。继续实施市级领导联系重点项目制度，全面推行“十不准”规定，从重从快处理不作为、低效作为、乱作为行为。优化施工环境。依法严厉打击强揽工程、强装强卸等扰乱正常施工的违法行为，依法妥善处理征地拆迁安置问题。切实解决群众的实际困难，争取群众对项目建设的理解、支持和配合。

　　（三）强力推进城市创建活动，加快新型城镇化步伐

　　强力推进创建全国文明城市、国家卫生城市、国家森林城市、国家交通管理模范城市工作，确保创建国家森林城市通过评估验收，力争创建国家卫生城市通过预评估，进一步提升城市的承载能力和吸纳能力，营造良好的人居环境。

　　抓好城乡规划。充分发挥规划在城乡建设中的龙头作用。抓好中心城区总体规划修编工作，完成控制性详规和专项规划的编制和审批。尽快启动统筹城乡规划编制工作。严格执行规划，提高规划的严肃性。坚决遏制违法建筑，规范房地产开发。

　　加快中心城区建设。加强中心城区道路、广场、公园等基础设施建设，继续抓好主次干道的提质改造。加强绿化、亮化等配套设施建设，抓好沿江两岸规划的实施及会龙山生态公园、清溪河城市湿地景观公园、银城大道绿化提质等重点工程。加强污水、垃圾处理设施建设，确保益阳高新区东部新区污水处理厂投入运行，力争城市垃圾焚烧发电项目开工。抓好城市资产经营，特别是经营好城市土地，确保市城建投发行16亿元企业债券，为城市建设筹措更多资金。加强建筑工程质量监管，确保工程质量安全。

　　加强城市管理。启动“智慧城市”的建设工作，加快“数字城管”建设，进一步推进精细化、规范化管理，着力形成长效管理机制。切实加强市政基础设施的维护管理，深入推进市容秩序专项整治行动，全面落实“门前三包五不准”责任制，进一步改善市容市貌，提升城市管理水平。切实推动管理重心下移，充分发挥街道、社区在城市管理中的重要作用。

　　大力推进小城镇建设。加强对城乡统筹试点镇草尾镇的规划指导，确定平口镇、灰山港镇等一批重点镇纳入市级规划调度范畴。加强村镇布局规划，提高乡村规划建设水平。加快基础设施和公共服务设施建设，提高小城镇综合服务能力和辐射带动能力。培育镇域经济支柱，增强小城镇经济实力。引导有条件的重点镇开展创建省级卫生县城（镇）工作，提升城镇管理水平。积极探索农民集中居住区建设，鼓励农民向城镇和农村新型社区集中，促进农村人口城镇化。

　　（四）强力推进城乡统筹，加快农业农村经济发展

　　加快发展现代农业。大力发展特色农业、规模农业、品牌农业。开展农村集体土地确权发证工作，每个区县（市）选择一个乡镇进行试点。创新和完善土地流转模式，在更多乡镇推行土地信托流转，进一步推动土地规模经营。加强农产品基地建设。深入开展水稻生产“三项行动”，大力发展蔬菜生产，抓好黑茶产业发展，加快药材产业发展。抓好生猪生产特别是标准化养殖，做大做强“中国淡水鱼都”。壮大农产品加工龙头企业，提高产品附加值。加强农业标准化建设，保证农产品质量安全。抓好农产品物流、农业科技创新、农业行政执法、农业机械化等相关工作。认真执行各项支农惠农政策，稳步推进各类农业政策性保险，大力发展农民专业合作组织，千方百计促进农民增收。

　　改善农村基础设施条件。加强农村路网建设。完成农村公路建设500公里，建好一批农村客运站场和客运招呼站，全面完成农村公路危桥改造任务。大力实施水利建设“十大工程”，完成年度建设任务；突出抓好农田水利和人饮安全项目建设，完成病险水库除险加固任务；认真实施洞庭湖综合治理近期项目，抓好大型灌排泵站更新改造、渠道清淤整治和水利血防工程建设。继续加强中小流域治理。推进新一轮农村电网改造升级工程，加强农村通信基础设施建设。深入推进农村清洁工程，继续抓好“四有两无清洁村”建设。积极探索中心村建设模式，加强中心村基础设施配套建设。

　　推进城乡公共服务均等化。着力改善农村办学条件，广泛开展“送教下乡”等活动，促进教育公平。加强乡镇文化站、村文化室等乡村文化设施建设。抓好乡镇卫生院、村卫生室建设，提高乡村医生补助标准。不断完善新型农村养老保险制度和新型农村合作医疗制度。继续大力推进大通湖区、益阳高新区东部新区、沅江草尾镇统筹城乡发展试验试点工作。

　　（五）强力推进改革开放，增强发展动力与活力

　　抓好重点领域与关键环节的改革。推进机关事业单位工资收入分配制度改革。进一步完善县乡财政管理体制改革。深化国库集中支付改革，积极推进财政预算公开。进一步加大税收协控联管工作力度，强化税收征管，提高信息管税、依法治税水平。稳步推进国有企业和国有资产监督管理体制改革，鼓励企业开展股权制度改革和股份制改造，充分发挥商协会在市场经济建设中的积极作用。深化医药卫生体制改革，提高农村卫生工作的管理水平。推进水务管理体制等“五项改革”。统筹抓好集体林权制度改革、基层农技推广体系改革、文化体制改革、教育管理体制改革、资源环境价费改革等。

　　大力推进金融创新。把金融创新作为激活要素的重中之重，确保信贷总量的快速增长，力争净增信贷投放80亿元以上。继续大力引进股份制商业银行，加快华融湘江银行益阳分行的筹建步伐。积极支持上海浦发银行在沅江市建立村镇银行。稳妥实施农村信用社改革，抓好益阳农村商业银行的组建工作。继续推进我市“金融安全区”建设，加强重点行业贷款风险监管，规范民间融资，打击非法集资，防范金融风险。充分发挥保险在社会风险防范和经济利益保障方面的重要作用。

　　进一步扩大招商引资。突出大项目招商。精心策划包装一批重大项目，主动对接大企业、大集团，着力引进战略投资者。突出产业招商。围绕重点产业，引进行业龙头和上下游配套企业，接长产业链条，增强产业竞争力。突出专业招商。进一步建立健全重点产业、重点项目、重点园区专业招商工作机制，提高招商引资项目的成功率。突出乡情招商。充分挖掘乡情资源，鼓励和支持益阳籍在外企业家回乡投资兴业。力争全年引进市域外资金300亿元。

　　加强对外交流与合作。抓住国际产业转移和生产要素流动加快的机遇，加快转变外贸方式，优化出口产品结构，扶持一批有发展潜力的出口企业，培育新的出口增长点。加强益阳海关机构和益阳商检分局等平台建设，进一步改善我市工业产品和农副产品的通关条件。加强与国外友好城市的往来，全面提升对外开放水平。

　　（六）强力推进绿色益阳建设，推动全面协调可持续发展

　　抓好节能减排工作。推行强制性节能标准，加强重点用能企业节能监管，坚决淘汰落后产能，完成省定节能降耗目标任务。积极推进行政机关、事业单位等公共机构和建筑、交通、商业、民用等领域的节能工作。加强主要污染物总量减排工作，重点抓好造纸、麻纺、化工、电力、建材和冶金等行业主要污染物排放总量控制。推进企业清洁生产，加快发展循环经济。

　　加强生态建设和环境保护。抓好国家现代林业示范市建设，广泛开展造林绿化，提高森林覆盖率和城市建成区绿化覆盖率。加强流域治理和生物多样性保护，重点抓好南洞庭湖湿地、国家和省级自然保护区、湿地公园、森林公园的建设和保护。加强矿产资源开发监管，推进绿色矿山建设。加强水源水体保护，加快城市中心区饮用水安全建设，推进县级以上行政区域水务一体化。进一步巩固砂石市场整治成果，加强河道管理、水资源管理和水土保持工作。加强农村环境保护与治理，加快推进农村环境连片整治，重点加强农村土壤和水域环境治理。严格执行“环境影响评价制度”和环境保护“一票否决制”。

　　倡导绿色文明。大力开展“绿色消费进万家”等主题宣传活动。倡导家庭绿色生活新模式，引导和鼓励消费无污染的物品，使用节能环保产品。积极开展无烟机关、无烟社区等创建活动。组织开展“绿色文明单位”、“绿色文明村镇”等评选活动。

　　全力推进“两型”社会建设。坚持把益阳东部新区建设作为建设绿色益阳和推进“四化两型”的重中之重，落实支持益阳东部新区创新发展、优先发展的政策措施，积极推进“十大改革”，全面启动鱼形山“两型”社会示范区基础设施和项目建设，基本完成区域3条主干道建设，启动鱼形山水库补水工程；全力推动区域合作开发，力争2－3个产业项目开工，开发建设取得实质性突破。

　　（七）强力推进为民办实事，着力构建民本益阳

　　全力为人民群众办实事。努力扩大就业再就业。以创建省级创业型城市为抓手，大力推动全民创业，全面落实支持创业、促进就业的政策措施；整合各类教育培训资源，有针对性地开展创业就业培训指导；加强公共就业服务，多渠道开发就业岗位，抓好就业援助，加快城乡劳动力转移就业。提高非公有制企业工资集体协商建制率，逐步提高最低工资标准，加强劳动监察执法和劳动人事争议调解仲裁，维护劳动者权益。加快建立城乡一体的社会保障体系。认真执行《社会保险法》，努力扩大养老、失业、医疗、工伤和生育保险覆盖面，实现城乡居民养老保险全覆盖，进一步提高城乡居民最低生活保障标准。加快残疾人社会保障体系和综合服务体系建设，大力发展社会福利和慈善事业，做好救灾救济和优抚安置工作。进一步加大保障性住房建设力度，抓好廉租房、公租房建设和农村危房改造、移民避险搬迁工作；加快城市棚户区、国有工矿棚户区、垦区危房和第二批国有林场危旧住房改造；努力扩大住房公积金覆盖面。着力实施食品药品安全放心工程，全面启动“放心猪肉”、“放心蔬菜”、“放心作坊”、“放心食堂”、“放心药店”等放心工程建设，严厉打击危害食品药品安全的违法犯罪行为。深入推进食品药品安全专项整治和城乡一体化建设，确保人民群众饮食用药安全。加强医药、教育、客运公交、出租车、住房等民生价格监管，维护正常市场价格秩序。抓住安化县纳入新一轮国家扶贫开发工作重点县和武陵山片区区域发展与扶贫攻坚规划试点县的机遇，进一步加大扶贫开发力度，抓好区县（市）对口帮扶安化县贫困乡镇工作。继续抓好移民后扶工作。加强法律援助工作。

　　全面发展社会事业。深入实施教育强市战略。切实加大教育投入，确保预算内教育经费占财政支出的比例同口径提高2个百分点；大力发展学前教育，加大合格学校建设力度，加强城市教育设施配套建设，在益阳高新区启动两所义务教育学校的新建工作，完成两所义务教育学校的改造任务；支持高等院校的建设与发展，推进职业教育培养模式改革，激发职业教育发展活力；着力提高教师队伍素质，全面提高教学质量；采取有力措施保障校园、校车安全。推进文化大发展大繁荣。大力实施“六个一”文化工程，加快建设文化强市。抓好城乡公共文化服务设施建设，提高公共文化服务水平；推进文化创新，繁荣文艺创作，加强文化保护与开发利用，增强文化发展活力；组织开展喜闻乐见、健康有益的群众性文体活动，丰富人民群众精神文化生活；加快发展文化产业，着力打造文化品牌，增强文化竞争力。办好第二届中国（湖南）黑茶文化节暨安化黑茶博览会。组织开展道德模范人物巡回演讲活动，促进全社会良好道德风尚的形成。加强公共卫生服务体系建设。扩大基本药物制度实施范围；抓好重大疾病防控和卫生应急工作；加强基层医疗卫生服务能力建设，不断提高基层医疗质量和医疗安全管理水平。继续稳定低生育水平，改善人口结构，提高人口素质。深入实施全民健身计划，提高竞技体育水平。加强耕地保护，抓好环洞庭湖基本农田建设重大工程，全面完成“数字益阳”地理空间框架建设。全面实施妇女儿童发展规划，保障妇女儿童权益。大力普及应急救护知识，认真开展初级救护培训。加快推进新一代天气雷达建设和农村气象灾害防御体系、农业气象服务体系建设。切实推进统计联网直报和企业一套表改革。加快广播电视事业发展。继续抓好档案、保密、民族、宗教、外事、侨务、台办、科协、地方志、人防、地震、水文、无线电管理等工作。

　　努力维护社会和谐稳定。积极推进“法治益阳”建设，深入开展法制宣传教育和依法治理工作。加强社会管理综合治理，落实维稳工作责任，抓好矛盾纠纷排查调处，推进人民调解、司法调解、行政调解“三调联动”，从源头上预防和减少不稳定因素。完善社会治安防控体系，从严打击暴力犯罪、经济犯罪、黑恶团伙、黄赌毒和盗抢等犯罪行为。加大用群众工作统揽信访工作的力度，进一步加强领导干部接访、信访积案化解工作，抓好信访接待中心和基层群众工作站建设，坚决减少赴省进京非正常上访。严格落实安全生产责任制，加强事故隐患排查和整治，打击非法生产经营，加强安全生产应急救援能力建设，杜绝重特大事故发生。完善突发公共事件应急处置机制，提高应急管理能力。加强国防动员和后备力量建设，支持国安、武警、消防部队建设。

　　（八）强力推进依法行政，切实加强政府自身建设

　　进一步推进民本政府建设。认真执行《湖南省政府服务规定》，推进服务型政府建设。把重视和改善民生作为一切工作的出发点和落脚点，以市长热线、信访、人大代表建议和政协委员提案办理为主要平台，进一步拓宽和老百姓的对话通道，认真解决和落实好老百姓关注的实际问题，让广大群众得到更多的实惠。进一步推进法治政府建设。认真贯彻落实《法治湖南建设纲要》和《湖南省行政程序规定》，全面推进依法行政。自觉接受人大及其常委会的法律监督、工作监督和政协的民主监督，认真落实人大及其常委会的决议决定和审议意见，提高人大代表议案、建议和政协委员提案办理质量。坚持重大问题与政协协商，积极听取各民主党派、工商联和无党派人士的意见和建议。加强社区等基层组织建设，促进居民（村民）自治。支持工会、共青团、妇联等群团组织依照法律和各自章程开展工作。进一步推进效能政府建设。科学合理地组织开展政府绩效评估。加强政务中心建设，推进政府信息公开和公共企事业单位办事公开，创建一批“政务公开示范点”和“公共企事业单位办事公开示范点”。建好政府外网平台，加快电子政务建设。进一步深化行政审批制度改革，继续精简和调整行政审批事项，优化行政审批流程，推行电子审批。加快转变政府职能，大力改进作风，增强公仆意识、服务意识，提高行政效能。进一步推进廉洁政府建设。加强对政府工作人员的教育管理，增强政府工作人员的廉政意识。进一步完善国有资产管理、工程建设、产权交易和政府采购等重要部门、重点领域和关键环节的行政监察和审计监督，加强公共资源交易中心建设。加强行政监察，健全和落实行政问责制及相关配套制度，加强对财政资金、预算使用和重大投资项目的审计监督。认真落实党风廉政建设责任制，切实加强惩治和预防腐败体系建设，以清正廉洁的政风推动各项工作。

　　各位代表，益阳发展充满希望，美好前景催人奋进。让我们在中共益阳市委的正确领导下，紧紧抓住历史机遇，勇敢面对各种挑战，开拓进取，扎实工作，努力让益阳人民生活得更加幸福、更有尊严！
